# Intrinsic Therapeutic Link between Recuperative Cerebellar Con-Nectivity and Psychiatry Symptom in Schizophrenia Patients with Comorbidity of Metabolic Syndrome

**DOI:** 10.3390/life13010144

**Published:** 2023-01-04

**Authors:** Jingyu Zhou, Xiao Guo, Xiaoli Liu, Yuling Luo, Xin Chang, Hui He, Mingjun Duan, Shicai Li, Qifu Li, Ying Tan, Gang Yao, Dezhong Yao, Cheng Luo

**Affiliations:** 1 The Clinical Hospital of Chengdu Brain Science Institute, MOE Key Lab for Neuroinformation, High-Field Magnetic Resonance Brain Imaging Key Laboratory of Sichuan Province, School of Life Sciences and Technology, University of Electronic Science and Technology of China, Chengdu 610056, China; 2 Department of Psychiatry, The Clinical Hospital of Chengdu Brain Science Institute, University of Electronic Science and Technology of China, Chengdu 610056, China; 3 Department of Neurology, The First Affiliated Hospital of Hainan Medical University, Haikou 570102, China; 4 The Key Laboratory for Computer Systems of State Ethnic Affairs Commission, Southwest Minzu University, Chengdu 610093, China; 5 Research Unit of Neuroinformation (2019RU035), Chinese Academy of Medical Sciences, Chengdu 610072, China

**Keywords:** schizophrenia, metabolic syndrome, fMRI, functional connectivity, cerebellum

## Abstract

Components of metabolic syndrome might be predictors of the therapeutic outcome of psychiatric symptom in schizophrenia, whereas clinical results are inconsistent and an intrinsic therapeutic link between weaker psychiatric symptoms and emergent metabolic syndrome remains unclear. This study aims to reveal the relationship and illustrate potential mechanism by exploring the alteration of cerebellar functional connectivity (FC) in schizophrenia patients with comorbidity metabolic syndrome. Thirty-six schizophrenia patients with comorbidity of metabolic syndrome (SCZ-MetS), 45 schizophrenia patients without metabolic syndrome (SCZ-nMetS) and 39 healthy controls (HC) were recruited in this study. We constructed FC map of cerebello-cortical circuit and used moderation effect analysis to reveal complicated relationship among FC, psychiatric symptom and metabolic disturbance. Components of metabolic syndrome were significantly correlated with positive symptom score and negative symptom score. Importantly, the dysconnectivity between cognitive module of cerebellum and left middle frontal gyrus in SCZ-nMetS was recuperative increased in SCZ-MetS, and was significantly correlated with general symptom score. Finally, we observed significant moderation effect of body mass index on this correlation. The present findings further supported the potential relationship between emergence of metabolic syndrome and weaker psychiatric symptom, and provided neuroimaging evidence. The mechanism of intrinsic therapeutic link involved functional change of cerebello-cortical circuit.

## 1. Introduction

Schizophrenia is a common, complex, heterogeneous cognitive syndrome that seems to originate from a disruption of brain development caused by genetic and/or environmental factors [1]. Antipsychotics as the main modality for treatment of schizophrenia, have provided a great improvement in the management of patients with schizophrenia, but virtually all antipsychotics have brought certain physical side effects [2,3]. The second generation or atypical antipsychotics present a set of adverse effects especially weight gain and metabolic disturbance [4,5,6]. Metabolic syndrome is the commonly used term for a cluster of clinical and metabolic factors, including central obesity, dyslipidemias, hypertension, and insulin resistance, which could increase the risk for type 2 diabetes mellitus, coronary artery disease and stroke [7]. Compared with healthy controls, higher prevalence rate of metabolic syndrome, up to 40.9–42.7%, in schizophrenia patients have been revealed in recent research [8]. Additionally, it is possible that the comorbidity of metabolic syndrome is associated with cognitive dysfunction of patients with schizophrenia, including executive function, attention, and auditory and verbal memory [9,10].

However, some studies found that components of metabolic syndrome might be predictors of the improvement in schizophrenia psychopathology [11,12,13,14]. Clozapine and olanzapine are the most evident antipsychotics that result in the metabolic dysregulation, including weight gain and hyperlipidemia. Additionally, the metabolic dysregulation in turn predicted the therapeutic efficiency of antipsychotics [15,16]. Treated with clozapine, the aggravation of metabolic disturbance was significantly associated with reduced scores of Positive and Negative Syndrome Scale (PANSS) in chronic patients [11,14,16]. In the meantime, a longitudinal study lasting 12 months in the first-episode patients also supported the relationship between emergent weight gain and psychopathology improvement [17]. Recently, a systematic review and network meta-analysis including 25,952 schizophrenia patients supported the existence of this association, whereas an intrinsic therapeutic link between symptom improvement and metabolic dysregulation was still unclear [12]. For this obscure relationship, the analysis of clinical unimodal data might be unable to draw a consistent conclusion. Multimodal neuroimaging by functional magnetic resonance imaging (fMRI), which has offered insightful information to exploration of pathological physiology in schizophrenia [18,19], could provide new insights into resolving the problem.

Cerebellum is a pivotal hub underlying pathological mechanism of schizophrenia [20]. In morphological studies, a meta-analysis revealed the significantly reduced gray matter volume of cerebellum in schizophrenia patients, suggesting cerebrum and cerebellum were collectively affected during development of schizophrenia [21]. Correspondingly, in functional neuroimaging studies, the disconnection hypothesis has been widely accepted as the core pathology of schizophrenia. Patients generally manifested disturbed functional coupling between cerebellum and multiple cerebral cortex regions [22,23]. The loss of FC in cerebello-cortical circuit observed in schizophrenia patients might generate psychiatric symptoms [24] and cognitive dysmetria [25]. According to our previous study [22], reduced grey matter in cerebellum acted as potential structural foundation of the functional disconnection. After the antipsychotics-assisted physical therapy, including transcranial magnetic stimulation and modified electroconvulsive therapy, restoration of network connectivity in cerebello-cortical circuit corresponded to amelioration of psychiatric symptoms of schizophrenia [26,27]. In the present study, we proposed that cerebello-cortical dysconnectivity as a persistent endophenotype of chronic patients with schizophrenia could participate in regulating the symptoms of schizophrenia. Alteration of cerebello-cortical FC pattern was assumed to be a possible mechanism of intrinsic therapeutic link between weaker psychiatric symptoms and emergent metabolic dysregulation.

The development of research on cerebellum supported functional heterogeneity of different cerebellar modules [28]. The studies from intrinsic connectivity network and task-based fMRI provided independent confirmation for the cerebellar sensorimotor cognitive dichotomy [23,28]. The cerebellar motor cluster comprises lobules V, VI, VIIb, and VIII. The cerebellar cognitive cluster comprises Crus I and Crus II [29]. In the current study, in order to explore the different roles of cerebellum modules playing in the pathology of schizophrenia, we followed our previous study to divide cerebellum into two modules for construction of cerebello-cortical FC [22].

In this study, we aim to evaluate the potential relationship between emergent metabolic syndrome and therapeutic outcome of schizophrenia and explore potential mechanism of therapeutic link from the viewpoint of neuroimaging in a sample including schizophrenia patients with comorbidity of metabolic syndrome (SCZ-MetS), schizophrenia patients without metabolic syndrome (SCZ-nMetS), and healthy controls (HC). We hypothesized that (1) SCZ-MetS group had weaker psychiatric symptom compared with SCZ-nMetS group; (2) The emergence of metabolic symptom correlated with lower psychiatric symptom score; Next, we constructed resting-state FC in cerebello-cortical circuit. We presumed that (3) the abnormal FC in cerebello-cortical circuit of patients compared with HC significantly improved in the SCZ-MetS group, underlying the severity psychiatry symptom. Finally, we explored the potential moderation effect of metabolic components on correlations between FCs and PANSS scores to confirm this relationship.

## 2. Materials and Methods

### 2.1. Participants

A total of 120 subjects were acquired in this study, including 36 schizophrenia patients with comorbidity of metabolic syndrome (SCZ-MetS) and 45 schizophrenia patients without metabolic syndrome (SCZ-nMetS) and 39 healthy controls (HC). All the chronic schizophrenia patients were recruited from the Clinical Hospital of Chengdu Brain Science Institute. The patients were diagnosed using the structured clinical interview for the DSM-IV axis 1 disorders-clinical version (SCID-I-CV), and all were being treated with medication. The illness duration of the patients was at least 2 years. The severity of psychiatric symptoms was evaluated using PANSS. HC were recruited from the local community through advertisements and word of mouth to participate in this study. HC were excluded based on current or past axis I disorder as verified using the Structure Clinical Interview for DSM-IV, history of neurological illness, traumatic brain injury, substance-related disorders, or first-degree relatives with history of psychosis. Four patients and four HC were excluded due to exceeded head motion. Thirty-four SCZ-MetS (44.42 ± 9.24 years old), 43 SCZ-nMetS (40.65 ± 12.24 years old) and 35 HC (39.49 ± 13.56 years old) were included in the final analysis. The Ethics Committee of the Clinical Hospital of Chengdu Brain Science Institute in accordance with the Helsinki Declaration approved this study. Written informed consent was obtained from each subject and his/her guardian before the participating in the study.

### 2.2. Imaging Data Acquisition

All magnetic resonance imaging (MRI) data were acquired on a 3T MRI scanner (GE DISCOVERY MR750, Waukesha, WI 53188, USA) in the Center of Information Medicine Research in University of Electronic Science and Technology of China. The head was fixed using foam pads and ears plugs to minimize the motion and scanning noise, respectively, during scanning. Resting-state brain fMRI was collected using gradient-echo EPI sequences (repetition time [TR] = 2000 ms, echo time [TE] = 30 ms, field of view [FOV] = 24 × 24 cm^2^, flip angle [FA] = 90°, matrix = 64 × 64, slice thickness/gap = 4 mm/0.4 mm). The brain fMRI scanning lasted for 510 s, and a total of 255 volumes were collected. The subjects were instructed to keep eyes open and look at the cross/plus on the screen. Subjects were also instructed to keep head motionless without falling asleep during the scanning.

### 2.3. Imaging Data Preprocessing

Functional imaging data were preprocessed using DPABI [30] and NIT toolboxes [31]. Firstly, the initial 5 volumes of imaging data were discarded. Following slice-timing correction and motion correction, the corrected images were normalized to the MNI space reslicing to 3 × 3 × 3 mm^2^ voxels. Then, image smoothing (FWHM 8 mm) was carried out. All participants whose maximum translation in any of the orthogonal directions larger than 2.5 mm or rotation larger than 2.5° were excluded from subsequent analysis. Then, six motion parameters and their first temporal derivative, white matter signal, cerebrospinal fluid signal were removed through linear regression. The global signal was not regressed out as had been recently suggested in processing the schizophrenia functional data [32]. Finally, fMRI data were passed through a band-pass 0.01 Hz–0.08 Hz.

### 2.4. Cerebello-Cortical Functional Connectivity Analysis

To assess the resting-state FC between cerebellum and cerebral cortex, we segregated cerebellum into motor and cognitive cerebellum modules according to our previous study [22]. The cerebellar cognitive cluster (CBCc) was defined as bilateral Crus Ⅰ and Crus Ⅱ. The cerebellar motor cluster (CBCm) was defined as the bilateral lobules Ⅴ, Ⅵ, Ⅶb, and VIII. The preprocessed blood oxygen level dependence (BOLD) time course in bilateral Crus Ⅰ and Crus Ⅱ were averaged to represent the functional time course of CBCc. Similarly, BOLD time course in bilateral lobules Ⅴ, Ⅵ, Ⅶb, and VIII were also averaged to represent the functional time course of CBCm. Pearson correlation coefficients were calculated between two cerebellar modules (CBCc and CBCm) and each of the voxel in cerebral cortex to obtain FC maps of each subject. The resulting values of FC maps were transformed to approximate a Gaussian distribution using Fisher’s r-to-z transformation. Thus, we obtained FC map of cerebello-cortical circuit for each subject.

### 2.5. Assessments of Metabolic Syndrome

All the components of metabolic syndrome and metabolic syndrome case history of patients with schizophrenia were collected in their medical histories, including height, weight, fast blood glucose (FBG), blood pressure (BP), triglyceride (TG), high-density lipoprotein (HDL), and treatment of metabolic disease. Given that total cholesterol (TC) and low-density lipoprotein (LDL) had been included in studies verifying associations between metabolic dysregulation and psychiatric symptoms [12,13,15,16], the two indexes of lipidemia were also included in this study. Body mass index (BMI) was calculated as the patient’s body weight in kilograms (kg) divided by his height in meters squared (m^2^). Systolic blood pressures (SBP) and diastolic blood pressures (DBP) were assessed by a digital blood pressure monitor. A peripheral venous blood sample was collected from patients. FBG level and liver function examination was performed for each individual patient. Lipid profile (TG, TC, HDL and LDL) was obtained from liver function examination.

For the definition of metabolic syndrome in this study, firstly, taking Asians’ ethnic specificity of relationship between BMI and body fat percentage into consideration [6], we lowered the BMI cut-off value in this study, from 30 kg m^−2^ to 27 kg m^−2^. Besides that, due to the standpoint that central obesity should be an “optional” rather than “essential” criterion for defining metabolic syndrome in an Asian cohort, whether the patients with schizophrenia were subdivided into SCZ-MetS group was decided according to a slightly altered definition based on diagnostic criteria proposed by International Diabetes Federation (IDF) in 2005 [33,34]. The patients with schizophrenia were diagnosed as metabolic syndrome if they displayed random three of the following criteria defined by IDF: (1) central obesity (BMI ≥ 27 kg/m^2^); (2) FBG ≥ 5.6 mmol/L or treatment for diabetes mellitus; (3) BP ≥ 130/85 mmHg or on anti-hypertensives; (4) TG ≥1.695 mmol/L or on lipid lowering agent; (5) HDL < 1.036 mmol/L for men, < 1.295 mmol/L for women or treatment for dyslipidemia with lipid-lowering agent.

### 2.6. Statistical Analysis

To detect the difference in psychiatric symptoms scores, medication dosage and illness duration between SCZ-MetS and SCZ-nMetS, PANSS scores, chlorpromazine equivalent and illness duration were compared using two-sample t-test. One-way analysis of Covariance analysis (ANCOVA) among SCZ-MetS, SCZ-nMetS and HC was performed with age and years of education as covariates to reveal difference of FC. Then, pairwise post hoc analysis was performed with age and years of education as covariates to assess difference of FC between individual groups. Moreover, in the pairwise post hoc analysis between SCZ-MetS and SCZ-nMetS, we appended medication dosage as a covariate to reduce the influence of drug factors on the results. In the post hoc analysis between two groups of patients and HC, the statistical threshold used was corrected using the false discovery rates (FDR) (*p* < 0.05). However, on account of less obvious differences between two groups of patients, we described findings of post hoc analysis between SCZ-MetS and SCZ-nMetS within the uncorrected statistical threshold (*p* < 0.005).

In order to explore the complex relationship among brain FC features, metabolic syndrome and psychiatric symptom, we performed the partial correlation analysis and moderation effect analysis in SCZ-MetS group, SCZ-nMetS group and two groups of patients (SCZ-MetS and SCZ-nMetS), respectively. Firstly, FC values of regions of interest (ROIs), as brain FC features, were extracted by creating corresponding spheres (radius: 3 mm) whose center is corresponding to coordinates of significantly different subregions according to pairwise post hoc analysis between SCZ-MetS and SCZ-nMetS. We obtained the following 5 ROIs: 3 for ROIs based on CBCc-cortical circuit (FC between left middle frontal gyrus and CBCc, FC between bilateral posterior precuneus and CBCc); 2 for ROIs based on CBCm-cortical circuit (FC between bilateral middle frontal gyrus and CBCm). Secondly, the partial correlation analysis was performed among FC values, indicators related to metabolic syndrome and PANSS scores in pairs with age, years of education and medication dosage as covariates. Thirdly, moderation effects of metabolic symptoms on the association between brain features and PANSS scores was performed using Hayes’s PROCESS macro (Model 1) [35] in SPSS ver. 26.0. Because of potential confounding influence on resting regional brain activity and connectivity [36], SBP and DBP were appended to the moderation effect analysis as a covariate.

## 3. Results

### 3.1. Demographics and Clinical Characteristics

Demographic information and clinical characteristics of three groups of subjects were presented in Table 1. No significant differences in age, gender, or years of education were observed. Additionally, no significant differences in medication dosage and illness duration was detected between SCZ-MetS and SCZ-nMetS. By comparison of psychiatric symptoms scores of PANSS, we found that the SCZ-MetS had lower positive symptom scores than SCZ-nMetS (t = −2.35, *p* < 0.05, *n* = 58). No group difference was observed in negative symptoms and general symptoms (Table 1).

The clinical characteristics of metabolic syndrome were presented in Table 2. Although there is no significant difference in low-density lipoprotein (LDL), total cholesterol (TC), systolic blood pressures (SBP), and diastolic blood pressures (DBP), SCZ-MetS group showed higher body mass index (BMI) (t = 3.63, *p* < 0.05, *n* = 68), fast blood glucose (FBG) level (t = 2.4, *p* < 0.05, *n* = 77), triglyceride (TG) level (t = 4.54, *p* < 0.05, *n* = 77) and lower high-density lipoprotein (HDL) level (t = −3.3, *p* < 0.05, *n* = 77) than SCZ-nMetS group.

**Figure 1:**
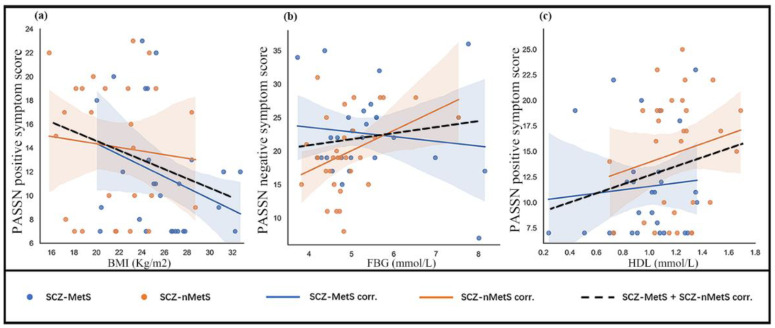
The association between symptoms of metabolic syndrome and severity of psychiatric symptoms. (**a**) Significantly inverse correlation between PANSS positive symptom score and BMI in SCZ-MetS group (r = −0.36, *p* = 0.039, *n* = 27) and all patients (SCZ-MetS group plus SCZ-nMetS group) (r = −0.249, *p* = 0.039, *n* = 55). (**b**) Significantly inverse correlation between FBG and PANSS negative symptom score in SCZ-MetS group (r = −0.345, *p* = 0.042, *n* = 28). (**c**) Significantly positive correlation between HDL level and positive symptom score in all patients (SCZ-MetS group plus SCZ-nMetS group) (r = 0.231, *p* = 0.043, *n* = 58). Abbreviations: BMI, body mass index; FBG, fasting blood glucose; HDL, high density lipoprotein.

Crucially, we observed inverse correlation between positive symptom and BMI in SCZ-MetS group (Figure 1a, r = −0.36, *p* = 0.039, *n* = 27) and in all patients (SCZ-MetS group plus SCZ-nMetS group) (Figure 1a, r = −0.249, *p*= 0.039, *n* = 55). FBG level was reversely correlated with negative symptom (Figure 1b, r = −0.345, *p* = 0.042, *n* = 28) in SCZ-MetS group. Finally, HDL level was positively associated with positive symptom (Figure 1c, r = 0.231, *p* = 0.043, *n* = 58) in all patients (SCZ-MetS group plus SCZ-nMetS group).

To explore the impact of medication dosage on metabolic components and psychiatric symptoms, correlation analysis between chlorpromazine equivalent and metabolic components, PANSS score was performed. No significant correlation was detected in all patients (SCZ-MetS plus SCZ-nMetS) (Figures S2 and S3). A summary medications information of all patients was added into Table S4.

### 3.2. Comparison of Cerebello-Cortical FC

The one-way analysis of Covariance analysis (ANCOVA) among SCZ-MetS, SCZ-nMetS, and HC on the FC between cortical regions and two cerebellar modules revealed a main effect of the diagnostic group in the extensive cortical region (Figure S1, Tables S1 and S2, false discovery rates (FDR) corrected, *p* < 0.05).

We found that FC between both modules of cerebellum and cerebral cortex represented the significant difference in primary motor cortex, primary somatosensory cortex, primary visual cortex and primary auditory cortex (Figure S1A,B, Tables S1 and S2, FDR corrected, *p* < 0.05). Compared to the motor module, the cognitive module exhibited more difference in the cerebral cortex related to the higher cognitive function, including bilateral insula and medial superior frontal gyrus.

**Figure 2:**
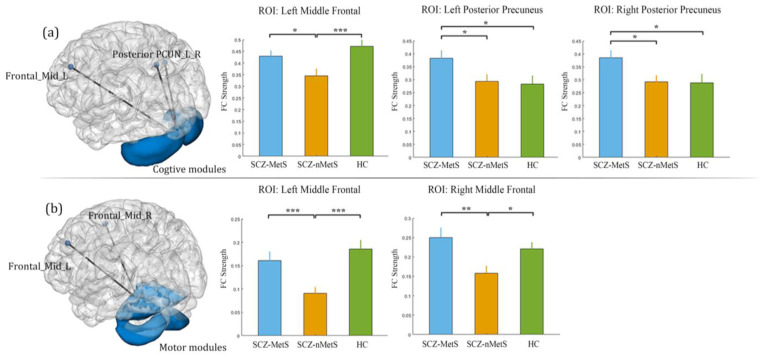
The post hoc t-test between SCZ-MetS group and SCZ-nMetS group. (**a**) SCZ-MetS group showed increased FC between cognitive module of cerebellum and bilateral posterior precuneus, left middle frontal gyrus. (**b**) SCZ-MetS group showed increased FC between motor module of cerebellum and bilateral middle frontal gyrus. Abbreviations: PCUN_L_R, bilateral precuneus; Frontal_Mid_L, left middle frontal gyrus; Frontal_Mid_R, right middle frontal gyrus. * *p* < 0.05; ** *p* < 0.01; *** *p* < 0.001.

The post hoc analysis showed that SCZ-MetS and SCZ-nMetS had significantly abnormal FC in cerebellar cognitive cluster (CBCc)-cortical circuits compared with HC, including abnormally increased FC within dorsal attention network (DAN), sensorimotor network (SMN), default mode network (DMN), salience ventral attention network (SVAN), visual network and abnormally reduced FC within DMN, DAN (Table S3, FDR corrected, *p* < 0.05; Table 3, FDR corrected, *p* < 0.05) based on the Schaefer atlas [37]. In particular, SCZ-nMetS showed increased FC between cognitive module of cerebellum and bilateral anterior precuneus and reduced FC between cognitive module of cerebellum and left middle frontal gyrus (CBCc-MFGL) compared with HC (Table 3, FDR corrected, *p* < 0.05). However, there is no significant difference in cerebellar motor cluster (CBCm)-cortical circuits (FDR corrected, *p* < 0.05) in SCZ-nMetS. The post hoc analysis between SCZ-MetS and SCZ-nMetS revealed that SCZ-MetS showed increased FC between cognitive module of cerebellum and bilateral posterior precuneus (Figure 2a, *p* < 0.05). Interestingly, the SCZ-MetS showed increased CBCc-MFGL FC (Figure 2a, *p* < 0.05). Additionally, SCZ-MetS also showed increased FC between CBCm and bilateral middle frontal gyrus (Figure 2b, *p* < 0.01).

### 3.3. Relationship between FCs and Metabolic Syndrome, Psychiatric Symptoms

**Figure 3:**
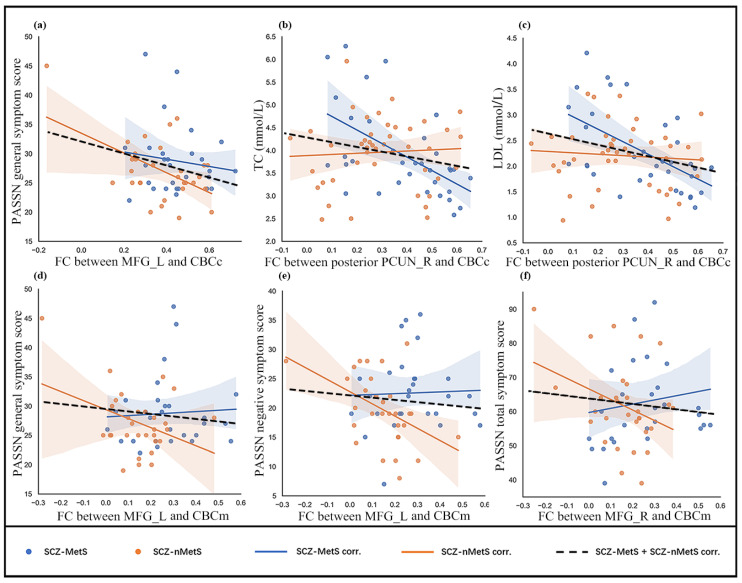
The association between brain features and metabolic syndrome, psychiatric symptoms in two groups of patients. (**a**) The negative correlation between CBCc-MFGL FC and PANSS general symptom score in all patients (SCZ-MetS group plus SCZ-nMetS group) (r = –0.299, *p* = 0.025, n = 58). (**b,c**) The negative correlations of FC between CBCc and right posterior precuneus with TC (r = –0.593, *p* < 0.001, n = 28, LDL (r = –0.588, *p* < 0.001, n = 28) in SCZ-MetS group. (**d,e**) The corre-lations between CBCm-MFGL FC and PANSS general symptom score (r = −0.45, *p* = 0.016, n = 30), PANSS negative symptom score (r = −0.493, *p* = 0.008, n = 30) in SCZ-nMetS group. (**f**) The corre-lation between CBCm-MFGR FC and PANSS total symptom score (r = −0.374, *p* = 0.05, n = 58) in SCZ-nMetS group. Abbreviations: MFG_L: right middle frontal gyrus; PCUN_R, right precuneus; MFG_R: right middle frontal gyrus; CBCm, cerebellar motor cluster; CBCc, cerebellar cognitive cluster.

Based on the post hoc analysis between SCZ-MetS and SCZ-nMetS, we chose 5 regions of interest (ROIs) (Figure 2a,b) for partial correlation analysis as described above. It revealed that CBCc-MFGL FC was negatively correlated with general symptom score in all patients (SCZ-MetS group plus SCZ-nMetS group) (Figure 3a, r= −0.299, *p*= 0.025, *n* = 58). Besides that, in SCZ-nMetS group, FC between CBCm and bilateral middle frontal gyrus (CBCm-MFGL and CBCm-MFGR) was negatively related to PANSS general symptom score (Figure 3d, r = −0.45, *p* = 0.016, *n* = 30), PANSS negative symptom score (Figure 3e, r = −0.493, *p* = 0.008, *n* = 30) and PANSS total symptom score (Figure 3f, r = −0.467, *p* = 0.014, *n* = 30). However, we did not find any significant relationship between FC of CBCc-MFGL, CBCm-MFGL, CBCm-MFGR and indicators of metabolic syndrome. For the FC between CBCc and right posterior precuneus, we observed significantly negative correlation between the FC and metabolic components including TC (Figure 3b, r = −0.593, *p* < 0.001, *n* = 28) and LDL (Figure 3c, r = −0.588, *p* < 0.001, *n* = 28) in SCZ-MetS group, which is not significant in SCZ-nMetS group. Finally, we did not find correlation of FC between CBCc and right posterior precuneus with psychiatric symptoms.

**Figure 4:**
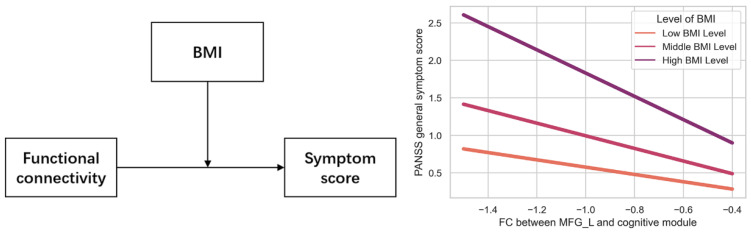
Moderation effect of BMI on association between FC of CBCc-MFGL and PANSS general symptom score in all patients (SCZ-MetS group plus SCZ-nMetS group) with schizophrenia. Abbreviations: BMI, Body mass index.; MFG_L, left middle frontal gyrus.

Using Hayes’s PROCESS macro (Model 1) [35] in SPSS ver. 26.0, all multiple regression models were constructed to examine the moderation effect of metabolic components on correlations between FCs of 5 ROIs and PANSS scores. Only the results of each metabolic symptoms’ moderation effect on association between FC of CBCc-MFGL and general psychiatric symptom were presented in Table 4. Although we did not find any association between FC of CBCc-MFGL and metabolic components, we observed moderation effect of BMI on association between CBCc-MFGL FC and general symptom score in all patients (SCZ-MetS group plus SCZ-nMetS group) (Table 4, Figure 4). We did not observe any more significant moderation effect in other multiple regression models.

## 4. Discussion

This study attempted to explore a therapeutic link between emergent metabolic dysregulation and weaker psychiatric symptom in schizophrenia. Besides that, we endeavored to reveal a possible mechanism of the therapeutic link by investigating FC pattern between cerebellum and cerebral cortex in patients with schizophrenia. We found weaker positive symptom in SCZ-MetS as well as significant correlations between metabolic disturbance, positive and negative symptom score of schizophrenia, which corroborated our hypothesis that the worse metabolic symptoms correlated with better therapeutic outcome of psychiatric symptoms. No significant correlation was found between medication dosage, metabolic components, and positive symptoms, which supported that larger dosages of antipsychotics could not fully explain the lower psychotic symptoms. Compared with SCZ-nMetS, SCZ-MetS showed different cerebello-DMN FC pattern. Importantly, CBCc-MFGL dysconnection of SCZ-nMetS could relieved in the SCZ-MetS. Finally, we observed moderation effect of BMI on association between CBCc-MFGL FC and general symptom score in all patients (SCZ-MetS group plus SCZ-nMetS group), supporting the moderating role of metabolic disturbance on relationship between FC and psychiatric symptom. These results had converged to suggest that cerebello-frontal circuit involved in mechanism of intrinsic therapeutic link between emergence of metabolic syndrome and weaker psychiatric symptom.

Whether an association exists between metabolic dysregulation and improvement of psychiatric symptom severity in patients with schizophrenia has not been completely determined, although some results with large cohort and meta-analyses have suggested that the most efficacious of antipsychotics, for example clozapine, might be associated with the most severe metabolic disturbance [12,38]. In the present study, no significant differences in medication dosage was detected between two subgroups of patients. However, PANSS positive symptom scores of SCZ-MetS was significantly lower than SCZ-nMetS, suggesting that larger dosages of antipsychotics could not fully explain the lower psychotic symptoms. Moreover, we found that some symptoms of metabolic syndrome, including increased BMI, FBG, and decreased HDL, were significantly correlated with reduce of PANSS scores. In clinical practice, metabolic dysfunction was associated with total psychiatric symptoms, especially positive symptoms and general symptoms [15,39]. BMI or weight gain might be the most frequent markers of favorable treatment outcome [11,15,17,40]. A previous longitudinal study recruiting first-episode schizophrenia patients supported that weight gain is related to antipsychotic efficacy in patients treated with flupenthixol decanoate over 12 months [1,17]. Olanzapine and clozapine, which were most frequently reported to show associations between therapeutic outcome and weight, were also two drugs with the most pronounced effects on glucose homeostasis [15]. Furthermore, pathoglycemia and dyslipidemia, such as elevation of FBG and reduction of HDL caused by antipsychotics, correlated negatively with change of PANSS scores in some studies [17,41,42,43]. Our findings may reflect a common clinical phenomenon that there is an association between emergent metabolic disturbance and weaker psychiatric symptom during the treatment for schizophrenia.

Cerebellum as a pivotal hub extensively connected with cortical regions [44,45]. Altered connectivity patterns between cerebellum and cerebral cortices could involve in pathogenesis of schizophrenia [27,46,47,48]. In our study, both SCZ-nMetS and SCZ-MetS showed significantly abnormal cerebello-cortical FC in cognitive module, including increased FC with regions in DAN, SMN, DMN, SVAN, VN and reduced FC in DMN, DAN. In line with our previous study [22,24], we revealed abnormal FC between cerebellum and cortical network. Increased cerebro-cerebellar connectivity has been observed in somatomotor networks in other seed-based studies [49]. Consistently, our findings observed abnormal FC in SMN, including dorsolateral superior frontal gyrus, supramarginal gyrus, precentral gyrus and postcentral gyrus, which could reflect cerebellum’s potential regulatory dysfunction on low-level sensorimotor system. Furthermore, patients showed increased FC between CBCc and SVAN including bilateral insula, which had also been supported by previous studies. In two independent cohorts of first-episode psychosis patients, increased seed-based FC between cerebellum and insula was believed to be involved with occurrence of auditory verbal hallucinations [50,51]. In addition, abnormal FC between Crus I of cerebellum and DAN, DMN in first episode psychosis patients commonly resonated our results that disordered FC in DAN and DMN [52]. In summary, we replicated previous results related to abnormal cerebello-cortical FC in patients with schizophrenia and extended these results in a module level.

Operation of DMN was considered the neural basis of spontaneous human thoughts and feelings [53]. Impaired cerebello-DMN interaction could contribute to the core feature of schizophrenia [54]. In our previous studies [22,24], abnormal FC between DMN and cerebellum was suggested to be a steady endophenotype of chronic patients with schizophrenia. Findings here also found detrimental cerebello-DMN FC in two groups of patients compared with HC. In addition, we further revealed the different cerebello-DMN connection pattern between SCZ-nMetS and SCZ-MetS. On the other hand, DMN also appeared to be especially vulnerable to metabolic abnormalities [55,56]. Disrupted resting state FC within DMN related to burden of vascular was implicated in cognitive function [56,57]. Overall, cerebellar-DMN FC could, respectively, underlie two possible patterns of mechanism: change of psychiatric symptom and adverse influence of metabolic syndrome on cerebral connectivity states.

For cerebello-DMN FC, results here have revealed that SCZ-MetS showed increased FC between cognitive module of cerebellum and bilateral posterior precuneus. Albeit there is no significant correlation between the increased FC and psychiatric symptoms, negative correlation with metabolic components was observed in SCZ-MetS group. Previous studies demonstrated that change of functional connectivity strength in precuneus and posterior cingulate was implicated with obesity-inducing and obesity-preventing behavior [58,59]. Besides that, reduced cerebral glucose metabolic rate in precuneus associated with insulin resistance could lead to the cognitive impairments [60]. The cerebellum generally acted as a purpose modulator to provide inhibiting feedback to the abnormal cortical activity [61,62]. Based on our findings and previous evidence, we speculated that abnormally increased FC between CBCc and posterior precuneus could present a compensation for impaired function of precuneus in SCZ-MetS.

Simultaneously, we found that all patients showed reduced CBCc-MFGL FC compared with HC. Importantly, both HC and SCZ-MetS showed increased CBCc-MFGL FC compared with SCZ-nMetS. Additionally, increase of FC in CBCc-MFGL was negatively correlated with reduction in PANSS general symptom scores in all the patients. Cerebello-cortical connectivity has been identified as the pivotal connectivity pattern determining severity of developmental mental disorders including schizophrenia [63]. Connectivity deficits between cerebellum and medial prefrontal cortex could predict conversion time and psychosis severity of clinical high-risk subjects and be associated with symptom severity of patients with schizophrenia [26,54,64,65]. There is a progressive reduction of cerebello-frontal FC with illness duration, with increased FC in early course patients and decreased FC in chronic patients, which is consistent with our study recruiting the chronic patients [47,52]. Therefore, our results demonstrated that dysconnectivity in CBCc-MFGL was a persistent endophenotype of chronic patients with schizophrenia. Moreover, findings here extend previous research insofar as patients with comorbidity metabolic syndrome recovered increased CBCc-MFGL FC compared with SCZ-nMetS. Meanwhile, we further found moderation effects of BMI in the correlation between CBCc-MFGL FC and general symptom score, indicating that the negative relationship tended to appear in patients with more severe weight gain. These results suggested a possibility that increased CBCc-MFGL FC might be a representation of symptom improvement in SCZ-MetS, which supported the link between worse metabolic dysregulation and better therapeutic outcome of psychiatric symptom from a viewpoint of neuroimaging.

For the relationship between metabolic syndrome and improvement of psychiatric symptoms of schizophrenia, there are some potential explanations. Firstly, Previous studies suggested that it was reasonable to hypothesize the existence of overlapping mechanism between therapeutic action and metabolic side effect of antipsychotics, in which insulin and 5-hydroxyptrytamine receptor type 2 (5-HT2) participate as a cross point [13,15]. Antipsychotics could impact brain insulin signal and induce peripheral insulin resistance [41,66]. Insulin as an important neuromodulator could affect neurotransmitters to regulate schizophrenia symptoms and change cerebello-cortical connectivity pattern [67,68]. Atypical antipsychotics as the antagonist of 5-HT2 could also influence the 5-hydroxytryptamine (5-HT) signal in central nervous system and peripheric tissue [62,69]. It has been revealed that not only did 5-HT2 participate in optimizing the rate of information flow from cerebellum by influencing the activation and suppression of Purkinje cells, but regulated adiponectin, lipolysis and gluconeogenesis [69,70]. The hypothesis of overlapping mechanism could explain the increase of CBCc-MFGL FC in SCZ-MetS. Secondly, polymorphism of cytochrome P450 enzymes determined metabolism of antipsychotics in liver and regulation metabolism of the local exogenous compounds in brain as well as synthesis of endogenous neuroactive substances (e.g., dopamine, serotonin). Individual polymorphism could represent different drug response and heterogeneous metabolic effect, which could resonate the moderation effect of BMI in current study. Finally, more detailed potential mechanism of this intrinsic therapeutic link between psychiatric symptom improvement and metabolic dysregulation need more research.

There are some limitations to the current study. Firstly, we did not collect complete clinical information for all the patients, including components of metabolic syndrome and PANSS scale scores. Soundly, though there is no significant difference in chlorpromazine equivalent between SCZ-MetS and SCZ-nMetS, we did not control the type of antipsychotics patients took. In addition, we did not control the influence of drugs to relieve metabolic syndrome such as lipid-lowering agents, which could conceal actual symptoms of metabolic syndrome. Lastly, the current study was a preliminary study to explore possible mechanism of intrinsic therapeutic link between weaker psychiatric symptom and emergent metabolic dysregulation, and many conclusions are still speculative. In future work, we tend to investigate this relationship in first-episode patients treated with single antipsychotics in a long-term longitudinal study.

## 5. Conclusions

In summary, we found CBCc-MFGL dysconnectivity of SCZ-nMetS was reconstructed in SCZ-MetS underlying weaker psychiatry symptom. The recuperative increased CBCc-MFGL FC in SCZ-MetS suggested a link between emergence of metabolic syndrome and better relieved psychiatric symptom in schizophrenia patients with comorbidity of metabolic syndrome. These results supported the potential relationship through multimodal neuroimaging and indicated that mechanism of intrinsic therapeutic link involved functional change of cerebello-cortical circuit. The current study offered new insights into clinical relationship between therapeutic benefit and metabolic health in schizophrenia.

## Figures and Tables

**Table 1:** Demographic information and clinical characteristics of three groups of participants.

Characteristic	SCZ-MetS (*n* = 34)	SCZ-nMetS (*n* = 43)	HC (*n* = 35)	Unadjusted
	Mean (SD)	Mean (SD)	Mean (SD)	*p*-Value
Gender (male/female)	27/7	28/15	26/9	0.199
Age (years)	44.42 (9.24)	40.65 (12.24)	39.49 (13.56)	0.205
Education (years)	11.94 (2.66)	11.42 (2.72)	12.14 (5.08)	0.657
Medication dosage (chlorpromazine equivalent)	337 (156)	320 (137)	-	0.683
Illness duration	20.3 (8.86)	17.2 (11.7)	-	0.2
PANSS total score	62.79 (14.11)	61.8 (12.32)	-	0.777
PANSS positive symptom score	11.5 (5.11)	14.87 (5.75)	-	0.022 *
PANSS negative symptom score	22.54 (6.37)	19.77 (5.94)	-	0.092
PANSS general symptom score	28.75 (5.99)	27.17 (5.37)	-	0.293

Note: we only collected 58 patients’ PANSS scale information (SCZ-MetS, (*n* = 28), SCZ-nMetS (*n* = 30)). Abbreviations: PANSS, Positive and Negative Syndrome Scale. * *p* < 0.05.

**Table 2:** Clinical characteristics of metabolic syndrome in patients with schizophrenia.

Characteristic	SCZ-MetS (*n* = 34)	SCZ-nMetS (*n* = 43)	Unadjusted
	Mean (SD)	Mean (SD)	*p*-Value
Body Mass Index	25.28 (3.6)	22.08 (3.63)	<0.001 ***
Fast blood glucose levels (mmol/L)	5.43 (1.106)	4.91 (0.7)	<0.05 *
Triglycerides (mmol/L)	1.59 (0.65)	0.99 (0.43)	<0.001 ***
Total cholesterol (mmol/L)	3.91 (0.96)	3.96 (0.76)	0.793
High-density lipoprotein (mmol/L)	0.996 (0.305)	1.22 (0.29)	<0.01 **
Low density lipoprotein (mmol/L)	2.28 (0.28)	2.2 (0.61)	0.561
Systolic blood pressure	117.81 (10.1)	116.38 (12.34)	0.71
Diastolic blood pressure	76.31 (7.22)	76.11 (7.7)	0.96

Note: we only collected 68 patients’ BMI index (SCZ-MetS, (*n* = 31), SCZ-nMetS (*n* = 37)). * *p* < 0.05; ** *p* < 0.01; *** *p* < 0.001.

**Table 3:** The difference of FC in CBCc and cerebral cortex in post hoc test between SCZ-nMetS and HC (FDR corrected, *p* < 0.05).

Cluster	Brain Region	MNI	T Value	Cluster Size (Voxels)	Brain Network
1	Left SFGmed	−1, 36, 30	−5.05	276	DMN
	Left SFG	−3, 27, 60	−3.13	\	DMN
2	Left ITG	−48, −9, −18	−4.88	113	DAN
3	Right ITG	51, −9, −39	−4.46	256	DAN
4	Left MFG	−36, 15, 60	−4.12	69	DMN
5	Right MFG	44, 14, 51	−3.16	49	DMN
6	Right PreCG	57, −15, 33	6.48	2884	SMN
7	Left PoCG	−54, −24, 51	6.27	1867	SMN
8	Left IPL	−59, −28, 43	5.36	230	DAN
9	Left MCC	−12, −15, 39	5.28	38	SVAN
10	Left LING	−6, −60, −3	5.10	172	VN
11	Right LING	6, −66, −3	5.07	155	VN
12	Left MOG	−30, −81, 18	4.92	262	VN
13	Right MOG	42, −75, −3	4.72	138	VN
14	Right Anterior PCUN	9, −42, 52	3.76	155	DMN
	Left Anterior PCUN	−10, −43, 52	3.40	\	DMN
15	Right INS	36, 10, 12	3.26	104	SVAN
16	Left INS	−30, 17, 12	3.22	137	SVAN

Abbreviations: SFGmed, medial superior frontal gyrus; ITG, inferior temporal gyrus; MFG, middle frontal gyrus; IPL, inferior parietal lobule; SOG, superior occipital gyrus; MTG, middle temporal gyrus; STG, superior temporal gyrus; PCUN, precuneus; PreCG, precentral gyrus; INS, insula; PCL, paracentral lobule; DMN: default mode network; DAN: dorsal attention network; VN: Visual network; SVAN: salience ventral attention network; SMN: sensorimotor network.

**Table 4:** The results of six moderate regressions of metabolic symptoms’ moderation effect on association between CBCc-MFGL FC and general psychiatric symptom.

Model	Dependent Variable	Independent Variable	*R2*	*F*	*p*	95%CI
Model 1	PASSN general symptom score	Intercept	0.07	4.30 *	0.84	[−0.24, 0.29]
		BMI			0.65	[−0.21, 0.34]
		FC			0.47	[−0.50, 0.23]
		BMI × FC			0.04	[−0.70, 0.01]
Model 2	PASSN general symptom score	Intercept	0.01	0.69	0.73	[−0.23, 0.32]
		FBG			0.77	[−0.22, 0.30]
		FC			0.09	[−0.64, 0.05]
		FBG × FC			0.41	[−0.24, 0.58]
Model 3	PASSN general symptom score	Intercept	0.01	0.70	0.84	[−0.25, 0.30]
		TG			0.66	[−0.34,0.22]
		FC			0.20	[−0.60, 0.13]
		TG × FC			0.41	[−0.20, 0.49]
Model 4	PASSN general symptom score	Intercept	0.001	0.03	0.79	[−0.23, 0.31]
		TC			0.16	[−0.50, 0.08]
		FC			0.29	[−0.67, 0.21]
		TC × FC			0.85	[−0.36, 0.44]
Model 5	PASSN general symptom score	Intercept	0.06	0.33	0.78	[−0.24, 0.31]
		HDL			0.67	[−0.40, 0.26]
		FC			0.13	[−0.62, 0.08]
		HDL × FC			0.57	[−0.49, 0.27]
Model 6	PASSN general symptom score	Intercept	0.003	0.1485	0.80	[−0.24, 0.30]
		LDL			0.17	[−0.51, 0.09]
		FC			0.35	[−0.65, 0.24]
		LDL × FC			0.70	[−0.30, 0.45]

* *p* < 0.05.

## Data Availability

The data supporting the findings of this study are available from the corresponding author upon reasonable request.

## Supplementary Materials

The following supporting information can be downloaded at: https://www.mdpi.com/article/10.3390/life13010144/s1.
