# Supplementary material for: Intrinsic Therapeutic Link between Recuperative Cerebellar Con-Nectivity and Psychiatry Symptom in Schizophrenia Patients with Comorbidity of Metabolic Syndrome
Source: Life (Basel). 2023 Jan 4;13(1):144. doi: 10.3390/life13010144 (PMC9863013; doi:10.3390/life13010144)
Supplement: Supplementary file 1 [file life-13-00144-s001.zip › life-2032053-supplementary.pdf]

Intrinsic therapeutic link between recuperative cerebellar connectivity and psychiatry symptom in schizophrenia patients with comorbidity of metabolic syndrome

Supplemental Information

Supplementary Figure

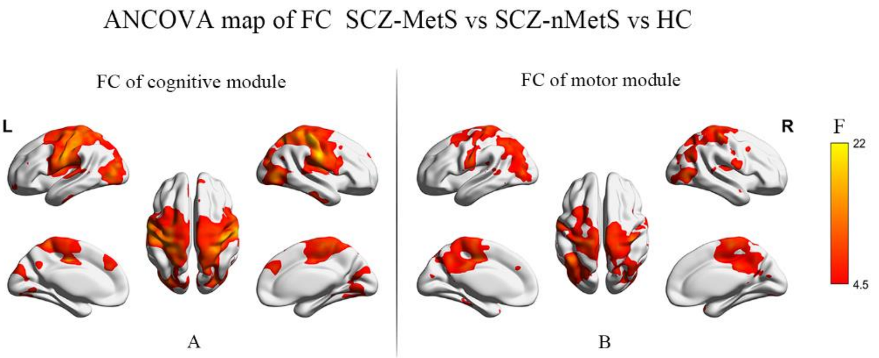

Figure S1. The results of ANCOVA among SCZ-MetS, SCZ-nMetS and HC on the FC between cortical regions and two cerebellar modules.

A) Difference of FC between cerebellar cognitive module and cortical regions. Significantly different regions included middle frontal gyrus, precuneus, insular lobe, primary motor cortex, primary somatosensory cortex, primary visual cortex and primary auditory cortex (FDR corrected, $p < 0.05$). B) Difference of FC between cerebellar motor module and cortical regions. Significantly different regions included middle frontal gyrus, precuneus, primary motor cortex, primary somatosensory cortex, primary visual cortex and primary auditory cortex (FDR corrected, $p < 0.05$).

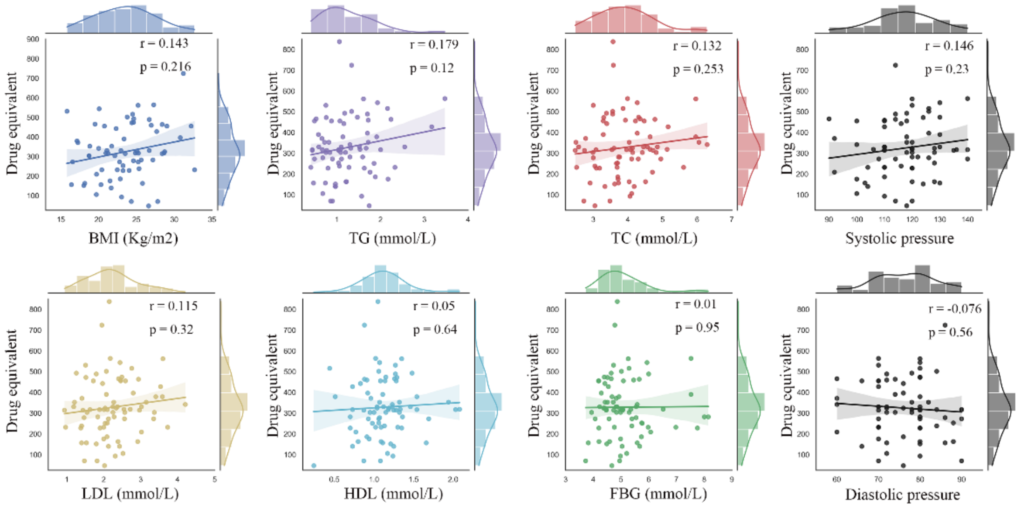

Figure S2. Impacts of medication dosage (chlorpromazine equivalent) on metabolic components. The correlation analysis between medication dosage and metabolic components. No significant correlation was found in all patients (SCZ-MetS plus SCZ-nMetS).

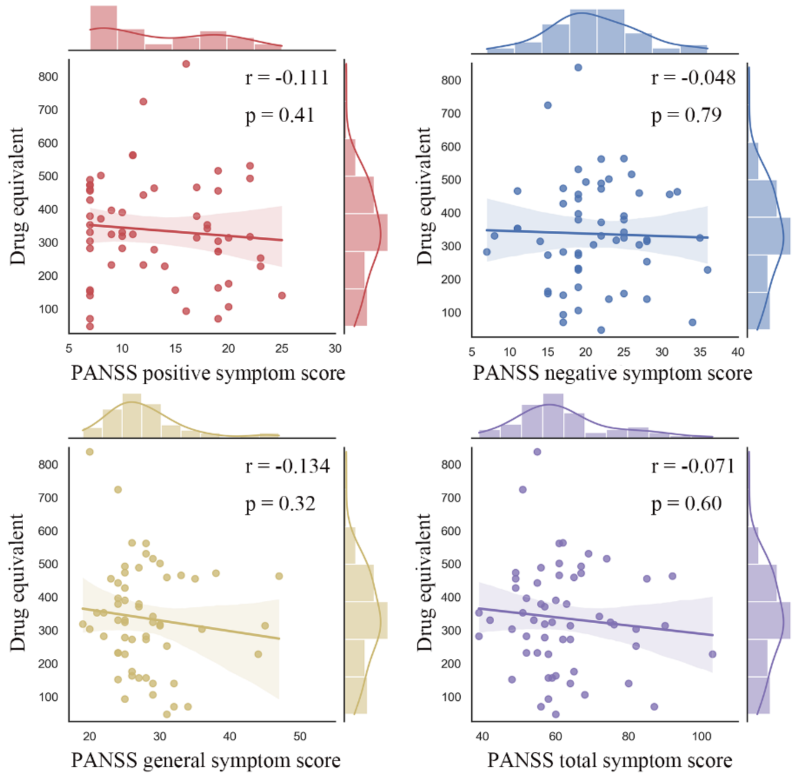

Figure S3. Impacts of medication dosage (chlorpromazine equivalent) on severity of psychiatric symptom. The correlation analysis between medication dosage and PANSS score. No significant correlation was found in all patients (SCZ-MetS plus SCZ-nMetS).

Supplementary Table

Supplementary Table S1

The difference of FC in CBCc and cerebral cortex in ANCOVA (FDR corrected, $p < 0.05$)

Cluster	Brain region	MNI	F value	Cluster size (voxels)	Brain Network
1	Right PoCG	60, –15, 30	22.36	1867	SMN
2	Left PreCG	–63, –12, 33	22.35	1765	SMN
3	Left MOG	–33, –81, 18	15.1	240	VN
4	Left ITG	–45, –12, –21	15.7	222	DAN
5	Left SFGmed	–1, 39, 30	14.75	52	DMN
6	Right INS	36, –6, 12	14.23	49	SVAN
7	Left MFG	–29, 60, 17	10.21	50	DMN
8	Left PCUN	–8, –45, 57	10	148	DMN

Abbreviations: PoCG, postcentral gyrus; PreCG, precentral gyrus; MOG, middle occipital gyrus; ITG, inferior temporal gyrus; SFGmed, medial superior frontal gyrus; INS, insula; MFG, middle frontal gyrus; PCUN, precuneus; SMN: sensorimotor network; VN: Visual network; DAN: dorsal attention network; SVAN: salience ventral attention network; DMN: default mode network.

Supplementary Table S2

The difference of FC in CBCm and cerebral cortex in ANCOVA (FDR corrected, $p < 0.05$)

Cluster	Brain region	MNI	F value	Cluster size (voxels)	Brain Network
1	Right MFG	18, 72, 0	16.34	102	DMN
2	Right PoCG	39, –27, 54	14.96	4573	SMN
3	Right MTG	69, –9, –18	13.67	39	SVAN

Abbreviations: MFG, middle frontal gyrus; PoCG, postcentral gyrus; MTG, middle tempral gyrus;

DMN: default mode network; DAN: dorsal attention network; SMN: sensorimotor network; SVAN: salience ventral attention network; VN: Visual network.

Supplementary Table S3

The difference of FC in CBCc and cerebral cortex in post-hoc test between SCZ-MetS and HC (FDR corrected, $p < 0.05$)

Cluster	Brain region	MNI	T value	Cluster size (voxels)	Brain Network
1	Left SFG	–30, 60, 15	–5.22	166	DMN
2	Right SFG	21, 72, 3	–4.75	217	SMN
3	Right ITG	60, –21, –24	–4.66	202	DAN
4	Right SFGmed	1, 39, 30	–4.56	209	DMN
5	Left ITG	–51, –12, –36	–4.35	170	DAN
6	Left PreCG	–33, –15, 63	6.41	6456	SMN
	Left IPL	–60, –25, 46	4.3	\	DAN
7	Left PoCG	–48, –18, 33	4.38	748	SMN
	Left PCUN	–8, –55, 38	2.89	\	DMN
8	Right INS	37, –6, 12	3.62	162	SVAN

Abbreviations: SFG, dorsolateral superior frontal gyrus; ITG, inferior temporal gyrus; SFGmed, medial superior frontal gyrus; PreCG, precentral gyrus; IPL, inferior parietal lobule; PoCG, postcentral gyrus; PCUN, precuneus; INS, insula;

DMN: default mode network; SMN: sensorimotor network; DAN: dorsal attention network; SVAN: salience ventral attention network;

Supplementary Table S4

Information on antipsychotic medication usage for each patient

Subject	Group	Drug1	Average dose (mg)	Drug2	Average dose (mg)	CPZ equivalent units	Number of drug type
Sub1	SCZ-MetS	Clozapine	325	Aripiprazole	5	378.8	2
Sub2	SCZ-MetS	Quetiapine	450	-	-	316.9	1
Sub3	SCZ-MetS	Clozapine	100	Quetiapine	600	515.1	2
Sub4	SCZ-MetS	Fluphenazine	6	-	-	340.9	1
Sub5	SCZ-MetS	Clozapine	175	Aripiprazole	15	395.7	2
Sub6	SCZ-MetS	Clozapine	50	Risperidone	6	501	2
Sub7	SCZ-MetS	Clozapine	125	Aripiprazole	20	427.3	2
Sub8	SCZ-MetS	Clozapine	325	Quetiapine	600	723.5	2
Sub9	SCZ-MetS	Clozapine	25	Risperidone	2	174.7	2
Sub10	SCZ-MetS	Clozapine	200	-	-	185.2	1
Sub11	SCZ-MetS	Clozapine	250	-	-	231	1
Sub12	SCZ-MetS	Risperidone	6	-	-	454.5	1
Sub13	SCZ-MetS	Clozapine	350	-	-	324	1
Sub14	SCZ-MetS	Clozapine	75	Ziprasidone	80	227.9	2
Sub15	SCZ-MetS	Clozapine	150	Risperidone	4	441.9	2
Sub16	SCZ-MetS	Clozapine	225	-	-	208.3	1
Sub17	SCZ-MetS	Clozapine	250	-	-	231	1
Sub18	SCZ-MetS	Clozapine	300	-	-	277.8	1
Sub19	SCZ-MetS	Clozapine	800	-	-	563.4	1
Sub20	SCZ-MetS	Clozapine	75	-	-	69.4	1
Sub21	SCZ-MetS	Quetiapine	400	-	-	281.7	1
Sub22	SCZ-MetS	Clozapine	100	Ziprasidone	120	330.2	2
Sub23	SCZ-MetS	Clozapine	200	Risperidone	4	488.2	2
Sub24	SCZ-MetS	Risperidone	4	Aripiprazole	10	458.8	2
Sub25	SCZ-MetS	Clozapine	75	-	-	69.4	1
Sub26	SCZ-MetS	Clozapine	50	-	-	46.3	1
Sub27	SCZ-MetS	Clozapine	150	Quetiapine	600	561.4	2
Sub28	SCZ-MetS	Quetiapine	400	-	-	287.1	1
Sub29	SCZ-MetS	Clozapine	500	-	-	463	1
Sub30	SCZ-MetS	Clozapine	25	Risperidone	3	250.4	2
Sub31	SCZ-MetS	Ziprasidone	120	-	-	237.6	1
Sub32	SCZ-MetS	Aripiprazole	10	-	-	155.8	1
Sub33	SCZ-MetS	Clozapine	100	Risperidone	5	471.4	2
Sub34	SCZ-MetS	Olanzapine	500	-	-	352.1	1
Sub35	SCZ-nMetS	Quetiapine	450	-	-	316.9	1
Sub36	SCZ-nMetS	Risperidone	4	-	-	303	1
Sub37	SCZ-nMetS	Quetiapine	450	-	-	316.9	1
Sub38	SCZ-nMetS	Clozapine	100	Ziprasidone	120	330.2	2

Sub39	SCZ-nMetS	Clozapine	175	Aripiprazole	10	317.8	2
Sub40	SCZ-nMetS	Clozapine	100	Ziprasidone	120	330.2	2
Sub41	SCZ-nMetS	Clozapine	100	-	-	92.6	1
Sub42	SCZ-nMetS	Clozapine	175	-	-	162	1
Sub43	SCZ-nMetS	Olanzapine	5	Clozapine	225	313.6	2
Sub44	SCZ-nMetS	Quetiapine	450	-	-	316.9	1
Sub45	SCZ-nMetS	Risperidone	3	-	-	227.3	1
Sub46	SCZ-nMetS	Aripiprazole	10	Fluphenazine	12	837.6	2
Sub47	SCZ-nMetS	Aripiprazole	25	-	-	389.4	1
Sub48	SCZ-nMetS	Quetiapine	500	-	-	352.1	1
Sub49	SCZ-nMetS	Haloperidol	5	-	-	271.7	1
Sub50	SCZ-nMetS	Haloperidol	5	-	-	271.7	1
Sub51	SCZ-nMetS	Aripiprazole	10	-	-	155.8	1
Sub52	SCZ-nMetS	Olanzapine	5	-	-	105.3	1
Sub53	SCZ-nMetS	Risperidone	7	-	-	530.3	1
Sub54	SCZ-nMetS	Olanzapine	10	Quetiapine	400	492.2	2
Sub55	SCZ-nMetS	Clozapine	150	Risperidone	1.5	252.5	2
Sub56	SCZ-nMetS	Clozapine	150	-	-	138.9	1
Sub57	SCZ-nMetS	Olanzapine	5	Clozapine	225	313.6	2
Sub58	SCZ-nMetS	Risperidone	4	-	-	303	1
Sub59	SCZ-nMetS	Haloperidol	5	-	-	271.7	1
Sub60	SCZ-nMetS	Clozapine	250	-	-	231	1
Sub61	SCZ-nMetS	Risperidone	6	-	-	454.5	1
Sub62	SCZ-nMetS	Clozapine	175	-	-	162	1
Sub63	SCZ-nMetS	Haloperidol	10	-	-	543.5	1
Sub64	SCZ-nMetS	Clozapine	100	Ziprasidone	120	330.2	2
Sub65	SCZ-nMetS	Clozapine	350	-	-	324	1
Sub66	SCZ-nMetS	Clozapine	400	-	-	370.4	1
Sub67	SCZ-nMetS	Olanzapine	5	Clozapine	225	313.6	2
Sub68	SCZ-nMetS	Risperidone	4	-	-	303	1
Sub69	SCZ-nMetS	Clozapine	175	Aripiprazole	10	317.8	2
Sub70	SCZ-nMetS	Risperidone	6	-	-	454.5	1
Sub71	SCZ-nMetS	Clozapine	325	Aripiprazole	5	378.8	2
Sub72	SCZ-nMetS	Risperidone	3	Ziprasidone	120	464.9	2
Sub73	SCZ-nMetS	Olanzapine	500	-	-	352.1	1
Sub74	SCZ-nMetS	Clozapine	150	-	-	138.9	1
Sub75	SCZ-nMetS	Clozapine	175	Aripiprazole	20	473.6	2
Sub76	SCZ-nMetS	Risperidone	2	-	-	151.5	1
Sub77	SCZ-nMetS	Risperidone	4	-	-	303	1

Supplementary Table S5

The list of primary abbreviations in this text

Characteristic	Abbreviations
Schizophrenia patients with comorbidity of metabolic syndrome	SCZ-MetS
Schizophrenia patients without metabolic syndrome	SCZ-nMetS
Healthy controls	HC
Positive and Negative Syndrome Scale	PANSS
Functional magnetic resonance imaging	fMRI
Functional connectivity	FC
Cerebellar cognitive cluster	CBCc
Cerebellar motor cluster	CBCm
Body mass index	BMI
Fast blood glucose	FBG
blood pressure	BP
Systolic blood pressures	SBP
Siastolic blood pressures	DBP
triglyceride	TG
high-density lipoprotein	HDL
total cholesterol	TC
low-density lipoprotein	LDL
Cognitive module of cerebellum and left middle frontal gyrus	CBCc-MFGL
Motor module of cerebellum and left middle frontal gyrus	CBCm-MFGL
Motor module of cerebellum and right middle frontal gyrus	CBCm-MFGR
